# Evidence in support of chromosomal sex influencing plasma based metabolome vs APOE genotype influencing brain metabolome profile in humanized APOE male and female mice

**DOI:** 10.1371/journal.pone.0225392

**Published:** 2020-01-09

**Authors:** Yuan Shang, Aarti Mishra, Tian Wang, Yiwei Wang, Maunil Desai, Shuhua Chen, Zisu Mao, Loi Do, Adam S. Bernstein, Theodore P. Trouard, Roberta D. Brinton

**Affiliations:** 1 Center for Innovation in Brain Science, University of Arizona, Tucson, Arizona, United States of America; 2 School of Pharmacy, University of Southern California, Los Angeles, California, United States of America; 3 Biomedical Engineering, University of Arizona, Tucson, Arizona, United States of America; 4 College of Medicine, University of Arizona, Tucson, Arizona, United States of America; Texas Technical University Health Sciences Center, UNITED STATES

## Abstract

Late onset Alzheimer’s disease (LOAD) is a progressive neurodegenerative disease with four well-established risk factors: age, APOE4 genotype, female chromosomal sex, and maternal history of AD. Each risk factor impacts multiple systems, making LOAD a complex systems biology challenge. To investigate interactions between LOAD risk factors, we performed multiple scale analyses, including metabolomics, transcriptomics, brain magnetic resonance imaging (MRI), and beta-amyloid assessment, in 16 months old male and female mice with humanized human APOE3 (hAPOE3) or APOE4 (hAPOE4) genes. Metabolomic analyses indicated a sex difference in plasma profile whereas APOE genotype determined brain metabolic profile. Consistent with the brain metabolome, gene and pathway-based RNA-Seq analyses of the hippocampus indicated increased expression of fatty acid/lipid metabolism related genes and pathways in both hAPOE4 males and females. Further, female transcription of fatty acid and amino acids pathways were significantly different from males. MRI based imaging analyses indicated that in multiple white matter tracts, hAPOE4 males and females exhibited lower fractional anisotropy than their hAPOE3 counterparts, suggesting a lower level of white matter integrity in hAPOE4 mice. Consistent with the brain metabolomic and transcriptomic profile of hAPOE4 carriers, beta-amyloid generation was detectable in 16-month-old male and female brains. These data provide therapeutic targets based on chromosomal sex and APOE genotype. Collectively, these data provide a framework for developing precision medicine interventions during the prodromal phase of LOAD, when the potential to reverse, prevent and delay LOAD progression is greatest.

## Introduction

APOE genotype and female chromosomal sex are two known risk modulators for late onset Alzheimer’s disease (LOAD), where APOE4 carriers [1–8] and females [9–14] exhibit a higher life-time risk of Alzheimer’s disease. An early indicator of LOAD risk is decline in brain glucose metabolism, a key phenotype observed during the prodromal phase of LOAD, and a hallmark of the disease [15–22]. APOE4 carriers exhibit lower brain glucose metabolism in early midlife [23–32], as do women during the perimenopausal endocrine transition, which is greatest in post-menopause [19, 33]. Further, females exhibit an earlier onset of brain glucose hypometabolic phenotype than males [33, 34].

Similarly, APOE4 carriers had an earlier onset of brain glucose hypometabolism in multiple brain regions, including posterior cingulate, parietal lobe, temporal lobe, prefrontal cortex, parahippocampal gyrus, and thalamus [24, 30], which was paralleled by a greater reduction in glucose metabolism in longitudinal analyses [23, 30]. Further, in both MCI and AD populations, APOE4 carriers display more widespread brain glucose hypometabolism [26] and a more severe phenotype in regions vulnerable to AD pathology, including parietal lobe, temporal lobe, and cingulate areas [27, 28, 35].

Mechanistic analyses indicate that brain glucose hypometabolism in AD models is associated with impairment in mitochondrial oxidative phosphorylation (OXPHOS) [36–43] which is also evident in perimenopausal females [44, 45] and in APOE4 carriers [31, 46, 47]. While previous studies using humanized APOE4 knock-in mice and isolated neurons confirmed down regulation of OXPHOS and energy metabolism genes compared to APOE3 counterparts [48–50], the impact of sex, and potentially sex–APOE interplay, is not fully understood. Further, given the high energy demand of the brain, glucose hypometabolism and mitochondrial bioenergetic dysregulation have a profound impact on the balance of brain metabolome. Brain proteomic analysis in humanized APOE4 knock-in mice revealed down-regulation of enzymes involved in metabolic processes from glycolysis, TCA cycle, amino acid metabolism, and to lipid metabolism [47]. Metabolomic analysis in AD patients also confirmed alternations in brain lipid profiles [51–53]. Mechanistic and clinical research revealed that brain lipid metabolic dysregulation is associated with white matter disintegration [54, 55], and can drive inflammatory responses in the brain to modulate the risk of LOAD [52, 56–58].

Herein, we describe a series of systems biology analyses that link peripheral and brain metabolic profiles in both females and males carrying humanized APOE 3 and 4 alleles (hAPOE3 and hAPOE4 respectively). We further report associations between neural structure, beta amyloid generation and peripheral and brain metabolomics and transcriptomic profiles. In this study, we investigated the impact of sex difference and APOE genotype on peripheral and brain metabolism in humanized APOE targeted replacement mice. We also investigated the impact of chromosomal sex and APOE genotype on brain biomarkers and imaging marker of AD in aged animals. Our transcriptomic analysis further supports lipid metabolism dysregulation as a susceptibility factor for AD, and present inflammation as a potential link and therapeutic target to prevent AD.

## Methods

### Animals

All animal studies were performed following National Institutes of Health guidelines on the use of laboratory animals and all protocols were approved by the University of Southern California Institutional Animal Care and Use Committee. Female and male humanized *APOE3* targeted replacement (hAPOE3) and *APOE4* targeted replacement (hAPOE4) homozygous mice on a C57BL/6 background strain were obtained from Taconic Inc. Male hAPOE3 (M.APOE3), male hAPOE4 (M.APOE4), female hAPOE3 (F.APOE3) and female hAPOE4 (F.APOE4) animals were aged to 16 months.

### Plasma metabolic markers

Fasting blood was collected by retro-orbital bleeding from both 6-month and 16-month-old mice into EDTA-coated blood tubes. Fasting glucose was measured by glucose meter (Abbott, 70804 and Abbott, 70819–70) on whole blood. Fasting plasma triglyceride level (Cayman Chemical, 10010303) and ketone body level (Cayman Chemical, 700190) were measured by colorimetric assays following manufacturer’s instructions. Fasting plasma insulin level was measured by ELISA assay (Crystal Chem, 90080) according to manufacturer’s instructions.

### Metabolomic analysis

Both plasma (fasted) and cortex samples (non-fasted) from each sex and APOE genotype group (N = 5) were sent to Translational Genomics Research Institute (Phoenix, AZ) for targeted metabolomic analysis using AbsoluteIDQ^®^ p180 kit (Biocrates Innsbruck, Austria). A total of 187 metabolites including amino acids, carnitines, sphingomyelins, phosphatidylcholine and lyso-phosphatidylcholine were queried using ultra-performance liquid chromatography-tandem mass spectrometry [(UP)LC-MS/MS]. All concentrations were scaled to sample weight (Cortex) or volume (Plasma). Missing values were replaced by synthetic minima (half of the minimum positive value, MetaboAnalyst [59]). Analytes were removed from analysis if they harbored >50% missing values. For PCA and heatmap visualization and statistical analysis, the concentration data were normalized by log2 transformation and autoscaling.

To compare potential Sex and APOE genotype effects, the metabolites were analyzed by Linear Mixed Effect Models through Limma 3.36.5 package [60], by specifying fixed effects: Gender + APOE + Gender*APOE.

### Brain perfusion

Animals were sedated with an injection of ketamine and xylazine. The animals were transcardially perfused with 4% paraformaldehyde for 5 minutes. Following which, the skull of the animal was detached and stored in Trump’s fixative (1% glutaraldehyde and 4% formaldehyde) at RT for 24 hours and then moved to 4°C until further processing. A total of animals, 2 animals/group were used.

### Magnetic resonance imaging (MRI)

Male and female, hAPOE3 and hAPOE4 mice were perfused with intact skulls underwent MRI imaging post-fixation using a 7T Bruker BioSpec^®^ preclinical MRI scanner. Anatomical 3-dimesional T2-weighted RARE (Rapid Acquisition and Refocused Echoes) images were collected with TR (Repetition time)/TE_eff_ (Effective Echo time) = 1500/40ms, RARE factor of 8, and 75μm isotropic resolution. In addition, three sets of Diffusion Magnetic Resonance Imaging (dMRI) were collected using 8-shot echo planar imaging with 32 directions and a diffusion weighting of b = 1000s/mm^2^, and 4 b = 0 images. In plane resolution was 150μm and slice thickness was 450μm. Three contiguous datasets shifted by 150μm, were collected such that super resolution reconstruction produced dMRI datasets with 150μm isotropic resolution.

### Magnetic resonance image analysis

The high-resolution structural MRI images were semi-automatically brain extracted using MRIcron (www.nitrc.org) and Mango (www.ric.uthscsa.edu/mango/) programs and bias field-corrected using N4 implemented in ANTs (www.nitrc.org). The data was further analyzed by registering a T2-weighted reference image and atlas with 356 regions of interest (ROIs) [61] to each animal using the SyN algorithm in ANTs. Regional brain volumes of hAPOE3 and hAPOE4, male and female mice were assessed and percentage normalized to their respective total brain volumes. Statistical analyses were conducted using student t-test.

Raw, low-resolution, dMRI images were motion and eddy-current corrected using FSL’s eddy-correct [62] and denoised using a diffusion-matched principal component analysis technique [63]. Subsequently, the three low resolution datasets were reconstructed using in-house super-resolution reconstruction software, written in Julia [64], to generate 150 μm isotropic dMRI data. The brain was then semi-automatically extracted from non-brain tissue, bias field corrected and run through a two-step SyN registration performed in ANTs to create a labeled atlas in individual diffusion space. The high-resolution dMRI data were then fit to the diffusion tensor imaging (DTI) model using weighted linear least squares [65]. From the DTI fit, fractional anisotropy (FA) was calculated on a voxel-by-voxel basis using in-house Python code. Parameter maps were analyzed by registering the mouse atlas to each individual fixed mouse brain dMRI data, and then comparing the mean value of the top quartile of FA in white matter ROIs. FA values from male and female hAPOE3 and hAPOE4 animals were statistically compared using Friedman’s non-parametric rank test.

### Dissection of the Brain

Mice were euthanized per NIH guidelines and IACUC animal protocol at University of Southern California. Following anaesthetization, animals were perfused with phosphate buffered saline before brain dissection. On ice, brainstem and cerebellum were first removed. The two hemispheres were then separated, and hippocampi were isolated from cortical tissue. Brain tissues were snap frozen in liquid nitrogen before being stored in -80°C for subsequent assays.

### RNA isolation

Frozen hippocampal tissues were directly homogenized in TRIzol^®^ Reagent (Invitrogen, 15596026) using The Bullet Blender^®^ and silicon beads. Chloroform was used to extract RNA from the homogenate at a volume ratio of 1:5 to that of the TRIzol^®^ Reagent. Ethanol was then used to precipitate nucleic acids from the aqueous phase. RNA was further purified using PureLink^™^ RNA Mini Kit (Invitrogen^™^, 12183018A) following manufacturer’s instructions. Purelink^™^ DNase (Invitrogen^™^, 12185010) was used to eliminate DNA contamination. Purified RNA was eluded in RNase-free, diH_2_O. RNA concentration and quality were checked by NanoDrop^™^ One.

### RNA sequencing (RNA-Seq)

RNA-Seq was conducted on bulk hippocampal RNA at Vanderbilt Technologies for Advanced Genomics (VANTAGE). Five animals were included per sex and APOE genotype group. Only RNA samples with an acceptable RNA quality indicator score (RQI >7) were used for sequencing. Sample was enriched for polyA-mRNA during library preparation. Sequencing was performed using Illumina HiSeq3000, with 75bp paired-end read length and 30 million read depth. Transcripts were mapped to mouse cDNA (ensembl release 95) using Salmon 0.91 [66].

Tximport V1.6.0 [67] was used to generate a counts table from salmon output and DESeq2 V1.18.1 [68] was used to calculate normalized read counts for each gene to perform expression analysis. DESeq2 uses a generalized linear model (GLM) to evaluate differential expression while accounting for biological variance and uses a Wald test statistic to evaluate significance. The GLM in the analysis is: ~ Sex + APOE + Sex*APOE. The fold change was determined by dividing the average normalized read counts of one group of samples over the other groups of samples for each gene. P-values were corrected using the Benjamini and Hochberg False Discovery Rate, total number of significantly differentially expressed genes (referred to as DEG, here and after) with p-adjusted values less than 0.05 was determined. For the heatmap of selected pathways, the average expression value of vst transformed normalized counts from DESeq2 is presented for each gene in the pathway.

### Gene set enrichment expression analysis (GSEA)

A gene set enrichment analysis (GSEA) was performed separately for each model using the ranked mRNA [69, 70]. The rank scores for differential gene expression were calculated from the lfcShrink function in DESeq2 [68] with a shrinkage type of “ashr” [71]. The obtained rank scores of each comparison were used to test for relationships between gene expression and different phenotypes using the GSEA preranked method based on the KEGG [72, 73] pathway gene sets and the p-value is based on 1000 permutations. Significantly perturbed metabolic pathways (p.adjust <0.05) were further detected.

### Principal component analysis

PCA analysis was performed to exclude potential outliers and evaluate the effect size of multiple factors. For the metabolite datasets, PCA was based on the normalized expression values. For RNA-Seq, PCA was based on VST transformed expression from DESeq2 [68]. In the PCA plot, each group is represented as the average position of all samples within the group and standard errors of each PCs as the error bars.

### Amyloid beta quantification

Frozen cortex samples were homogenized with Tissue Protein Extraction Reagent (T-PER) (Thermo Scientific, Cat # 78510) using the Bullet Blender (Next Advance, Cat # BBX24B). Protein concentrations were determined by using the BCA protein assay kit (Pierce, Rockford, IL). Amyloid beta 40 and 42 concentrations were assessed using MSD Aß peptide Panel 1 kits 4G8 (MSD, K15199E) with equal amount of protein (100 μg/ 25 μl) for each sample following the manufacturer’s instructions.

### Statistical analysis

Statistical significance between groups was determined by unpaired t-test. For comparison of diffusion tensor metric- fractional anisotropy, use of Friedman’s non-parametric rank test was conducted. Significance was defined as p<0.05.

## Results

### Impact of sex and APOE genotype on blood based metabolic indicators

Change in metabolic profile is among the earliest indicators of risk AD [19–21, 25, 30, 33, 74] and also play an essential role in early AD mechanisms especially in white matter degeneration [54, 75]. To access the impact of sex and APOE genotype on peripheral metabolic profile, fasting plasma levels of glucose, insulin, triglyceride and ketone bodies, were quantified in hAPOE3 and hAPOE4, male and female mice at 6 and 16 months old.

**Fig 1:**
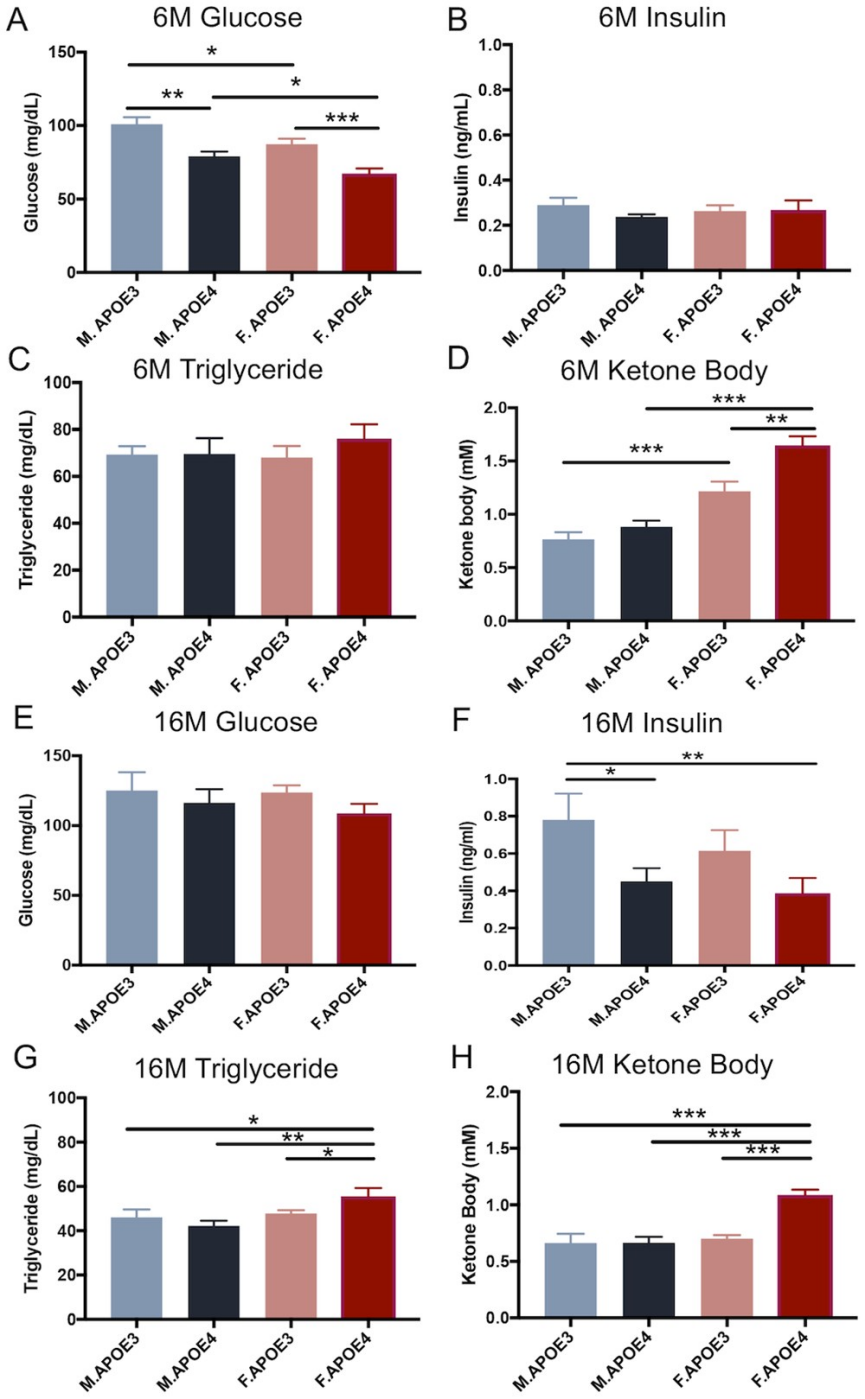
Fasting plasma levels of metabolic indicators. Fasting plasma levels of metabolic indicators of APOE3 and APOE4, male and female mice were measured at 6 months (A-D) and 16 months (E-H) old. At 6 months of age, (A) male mice had significantly higher fasting glucose levels compared to their female counterparts with the same APOE genotype. In both sexes, APOE3 mice have significant higher fasting glucose levels comparing to their APOE4 counterparts. The insulin level (B) was highest in APOE3 males whereas the triglyceride level (C) was highest in APOE4 females, though not significant. (D) Female mice exhibited significantly higher fasting ketone body level compared to their male counterparts with the same APOE genotype. APOE4 females also had significantly higher ketone body level compared to APOE3 females. At 16 months of age, no significant differences were observed in glucose levels across these four groups (E). However, APOE3 males exhibited significantly higher insulin levels (F) compared to APOE4 males and females. APOE4 females exhibited significantly higher triglyceride (G) and ketone body (H) levels compared to the other three groups. All data are presented as mean ± SEM, * P< 0.05, ** P< 0.01, *** P< 0.001.

At 6 months of age, male mice exhibited significantly higher fasting glucose levels compared to their female counterparts with the same APOE genotype (Fig 1A). In parallel, hAPOE3 mice exhibited significantly higher fasting glucose levels compared to their hAPOE4 counterparts of same sex. In contrast, female mice exhibited significantly higher fasting ketone body levels (Fig 1D, N = 10) compared to their male counterparts with the same APOE genotype. Further, hAPOE4 females exhibited significantly higher ketone body level compared to hAPOE3 females. In parallel, though not significant, insulin blood level was highest in hAPOE3 males (Fig 1B, N = 10) whereas the triglyceride level was highest in hAPOE4 females (Fig 1C, N = 10).

At 16 months of age, no significant differences were observed in glucose levels between males and females or between APOE genotypes (Fig 1E, N = 7–13). However, hAPOE3 males exhibited significantly higher insulin level compared to hAPOE4 males and females (Fig 1F, N = 7–13). hAPOE3 females also exhibited higher insulin levels, though not significant, compared to hAPOE4 females. In contrast, hAPOE4 females exhibited significantly higher triglyceride and ketone body levels compared to the hAPOE3 females and hAPOE3 and hAPOE4 males (Fig 1G, N = 7–13; Fig 1F, N = 7–13).

Together, both chromosomal sex and APOE genotype were associated with specific peripheral metabolic profiles that were most pronounced at 16 months of age. These peripheral metabolic profiles were associated with higher insulin levels in hAPOE3 males and higher triglyceride and ketone body levels in hAPOE4 females.

### Impact of sex and APOE genotype on plasma metabolic profile

To expand the characterization of sex and APOE genotype impact on peripheral metabolism and to parallel analyses conducted in the human brain [76, 77], fasting plasma levels of additional 188 targeted intermediates or final metabolites of key biochemical pathways, including amino acid, acylcarnitine, sphingomyelin and phosphatidylcholines, were measured in 16 months old hAPOE3 and hAPOE4, male and female mice using AbsoluteIDQ-p180 metabolomics panel.

**Fig 2:**
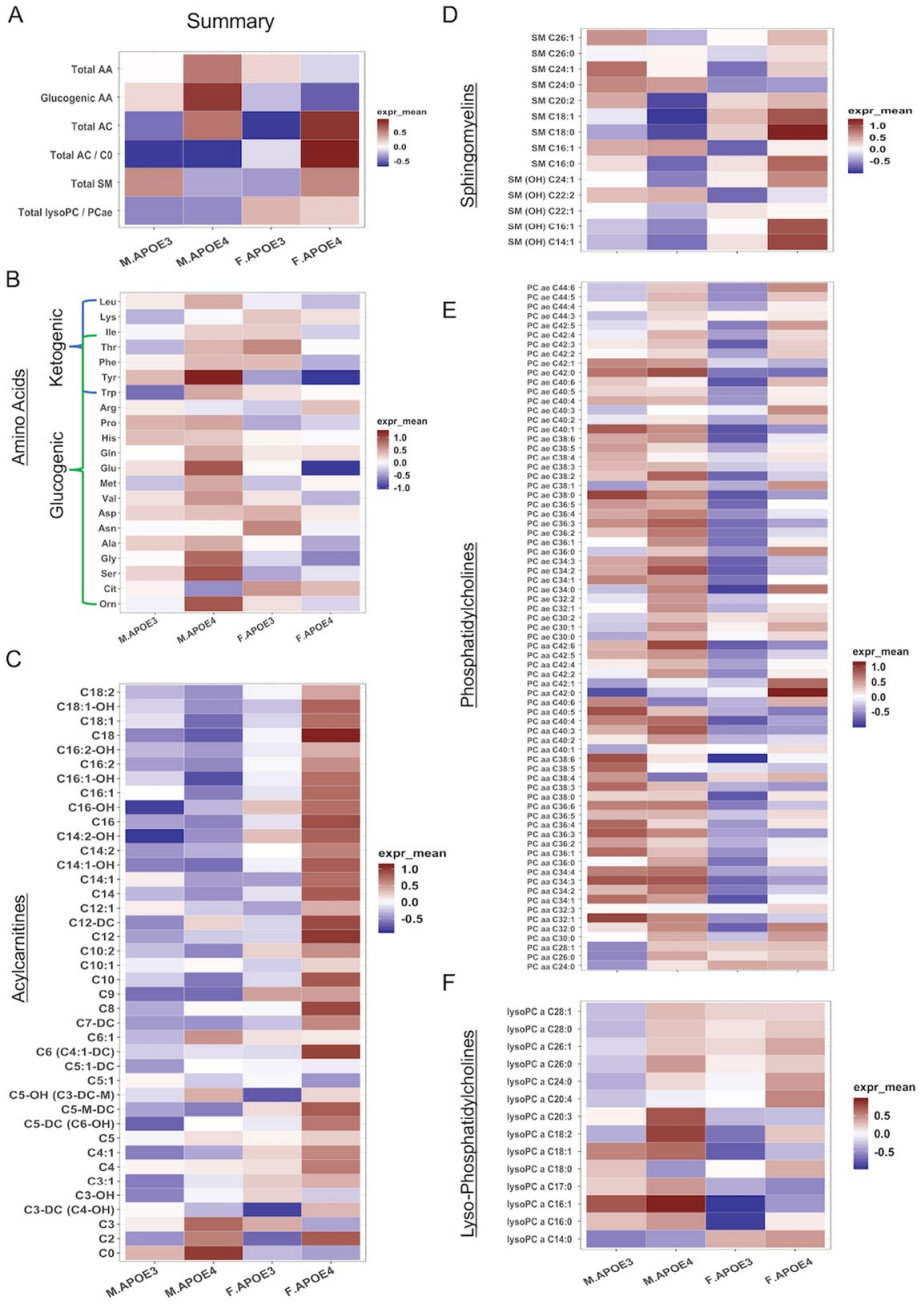
Plasma metabolomic profile: Differential regulation by sex and APOE genotype of peripheral metabolites. (A) Total plasma levels of major metabolite group of 16 months old APOE3 and APOE4, male and female mice. Amino acid (B) levels were higher in APOE4 males. Acylcarnitine (C) and sphingomyelin (D) levels were higher in APOE4 females. Phosphatidylcholine (E) levels were higher in males, while lysophosphatidylcholine(F) levels were higher in both APOE4 male and female mice.

Consistent with peripheral metabolic indicators, AbsoluteIDQ-p180 metabolomics outcomes indicated that male mice, regardless of their APOE genotype, were characterized by an amino acid metabolomic profile, as indicated by higher glucogenic amino acid levels (Fig 2A and 2B). In contrast, female mice, regardless of their APOE genotype, were characterized by a lipid metabolomic profile, indicated by the relative higher acylcarnitine levels (Fig 2A and 2C). The presence of hAPOE4 allele further exaggerated the sex-specific difference in metabolism and resulted in higher amino acids levels in hAPOE4 males and higher acylcarnitine levels in hAPOE4 females when compared to their hAPOE3 counterpart.

Sphingomyelins (SM) are critical components of the cell membrane, and are especially enriched in the myelin sheath surrounding neural axons. Dysregulated SM metabolism has been reported in AD patients and is involved in synaptic dysfunction [78, 79]. As shown in Fig 2D, hAPOE4 females exhibited the highest plasma sphingomyelin levels, especially SM(OH), when compared to the other three groups, suggesting a combined sex and hAPOE4 genotype effect on SM metabolism.

Phosphatidylcholines (PCs) are key components of neural membranes and have been reported to be involved in AD pathology [78]. Plasma PC levels were higher in males relative to females and were particularly elevated in hAPOE4 males (Fig 2E). A comparable relative sex difference was apparent in male lysoPC levels with hAPOE4 males exhibiting the highest level (Fig 2F). Relative to males, lysoPC was lower in both hAPOE3 and hAPOE4 females with hAPOE4 females exhibiting an increase compared to their hAPOE3 counterparts (Fig 2F). These data are consistent with both sex and APOE genotype differences in PC metabolism (Fig 2E and 2F).

Collectively, peripheral metabolic indicators coupled with plasma-based metabolomics indicate unique sex differences with males showing a greater propensity towards a glucose metabolic profile whereas females show a greater propensity towards a lipid metabolic profile. The impact of the APOE4 genotype on the metabolic phenotype in both sexes appears to exaggerate the existing sex-based profile, heightening the glucose profile in males and the lipid profile in females.

### Impact of sex and APOE genotype on brain metabolomics profile

To advance translational validity of discovery research, the interaction of sex and APOE genotype on metabolomic profiles was analyzed in the cerebral cortex from 16 months old hAPOE3 and hAPOE4, male and female mice using AbsoluteIDQ-p180 metabolomics to parallel analyses conducted in the human brain [76, 77].

**Fig 3:**
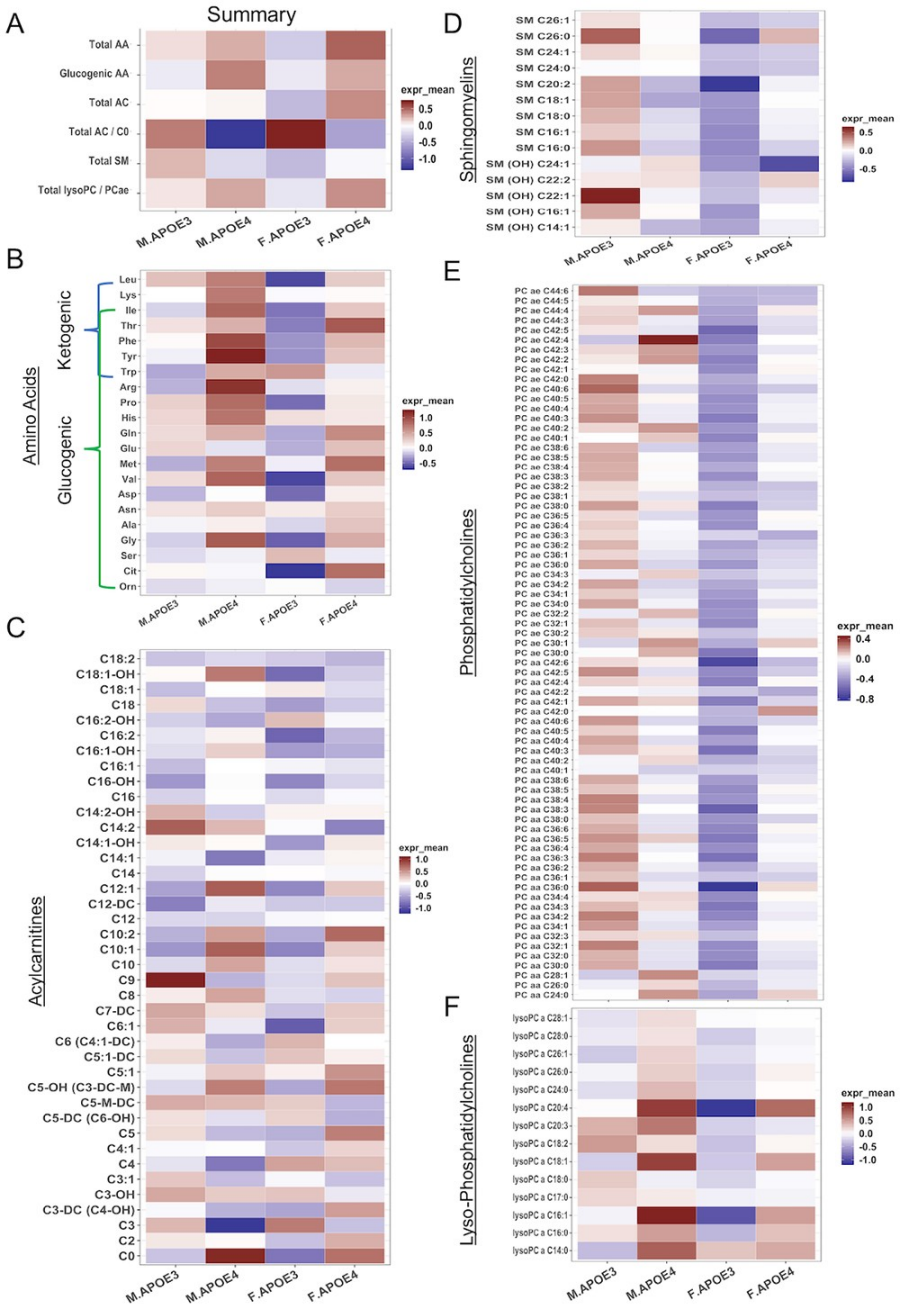
Cortex metabolomic profile: Differential regulation by sex and APOE4 genotype of brain metabolites. (A) Total cortical levels of major metabolite groups of 16 months old APOE3 and APOE4, male and female mice. Cortical levels of amino acid (B), carnitine (C) and lysophosphatidylcholine (F) were higher in APOE4 males and females. APOE3 males exhibited higher sphingomyelin (D) and phosphatidylcholines (E) levels than the other three groups.

Outcomes of metabolomics analyses indicated that in both sexes, cortical levels of amino acids (Fig 3A and 3B), carnitine (Fig 3A and 3C) and lysoPCs (Fig 3A and 3E) were higher in hAPOE4 males and females when compared to their hAPOE3 counterparts. In contrast, hAPOE3 males exhibited the highest SM (Fig 3A and 3D) and PC (Fig 3A and 3E) levels than the other three groups. In addition to APOE genotype effect, hAPOE3 and hAPOE4 males exhibited an increased cortical amino acid and lysoPC levels compared to their female counterparts (Fig 3B and 3F).

**Fig 4:**
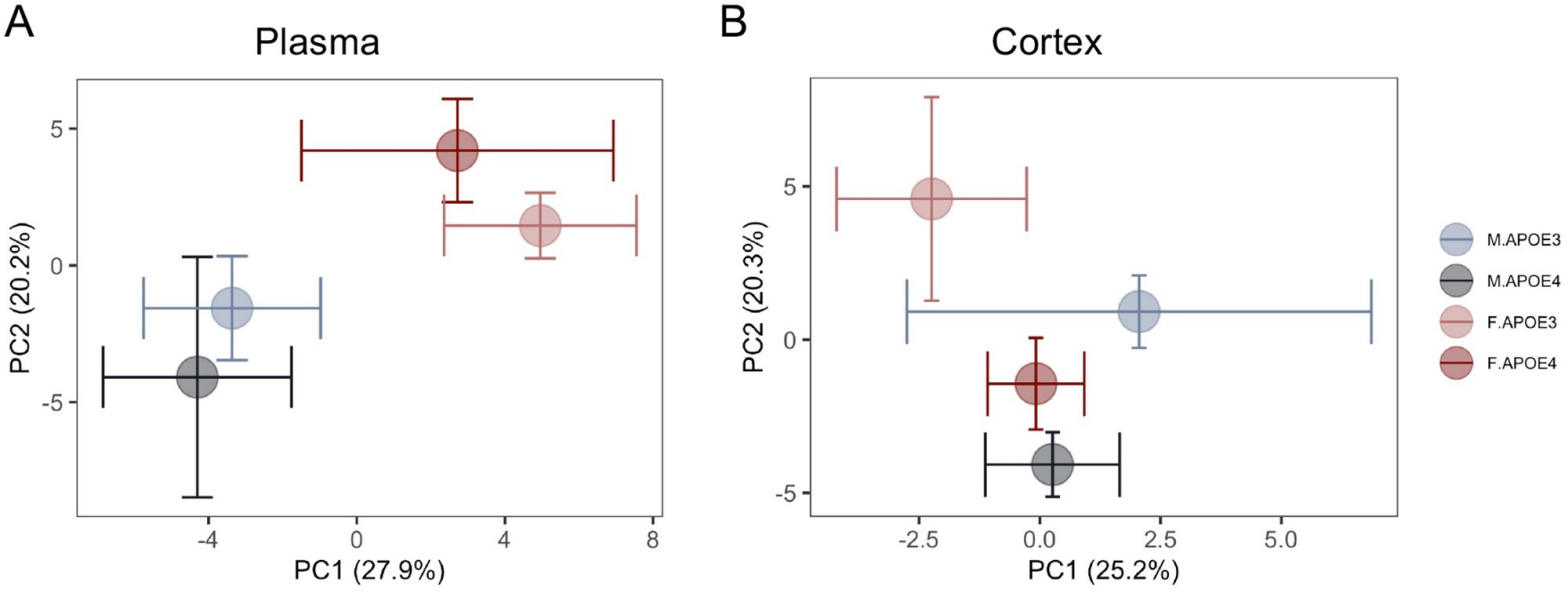
Principal component analysis of plasma and brain metabolomic profile: Chromosomal sex separation on plasma metabolome vs APOE genotype separation on brain metabolome. (A) The clustering pattern of plasma metabolites from 16 months old APOE3 and APOE4, males and females were primarily separated by sex and then by APOE genotype in females only. (B) In contrast, the clustering pattern of brain metabolites were well separated by sex and APOE genotype in APOE3 carriers only whereas APOE4 males and females showed no separation.

Principal component analysis (PCA) further indicated that in contrast to the more dominant sex effect observed on plasma metabolomic profiles, the effect of APOE genotype was more apparent in brain metabolomic profiles. As shown in Fig 4A, the clustering pattern of plasma metabolites from APOE genotype groups were well separated by sex and not by APOE genotype, suggesting a primary sex effect on peripheral metabolic profile. In contrast, the clustering pattern of brain metabolites from APOE genotype groups were well separated by both APOE genotype and sex (Fig 4B). These results indicated that the major APOE genotype-associated metabolomic changes are most evident in brain.

### Sex and APOE genotype difference in transcriptome of metabolic pathways

**Fig 5:**
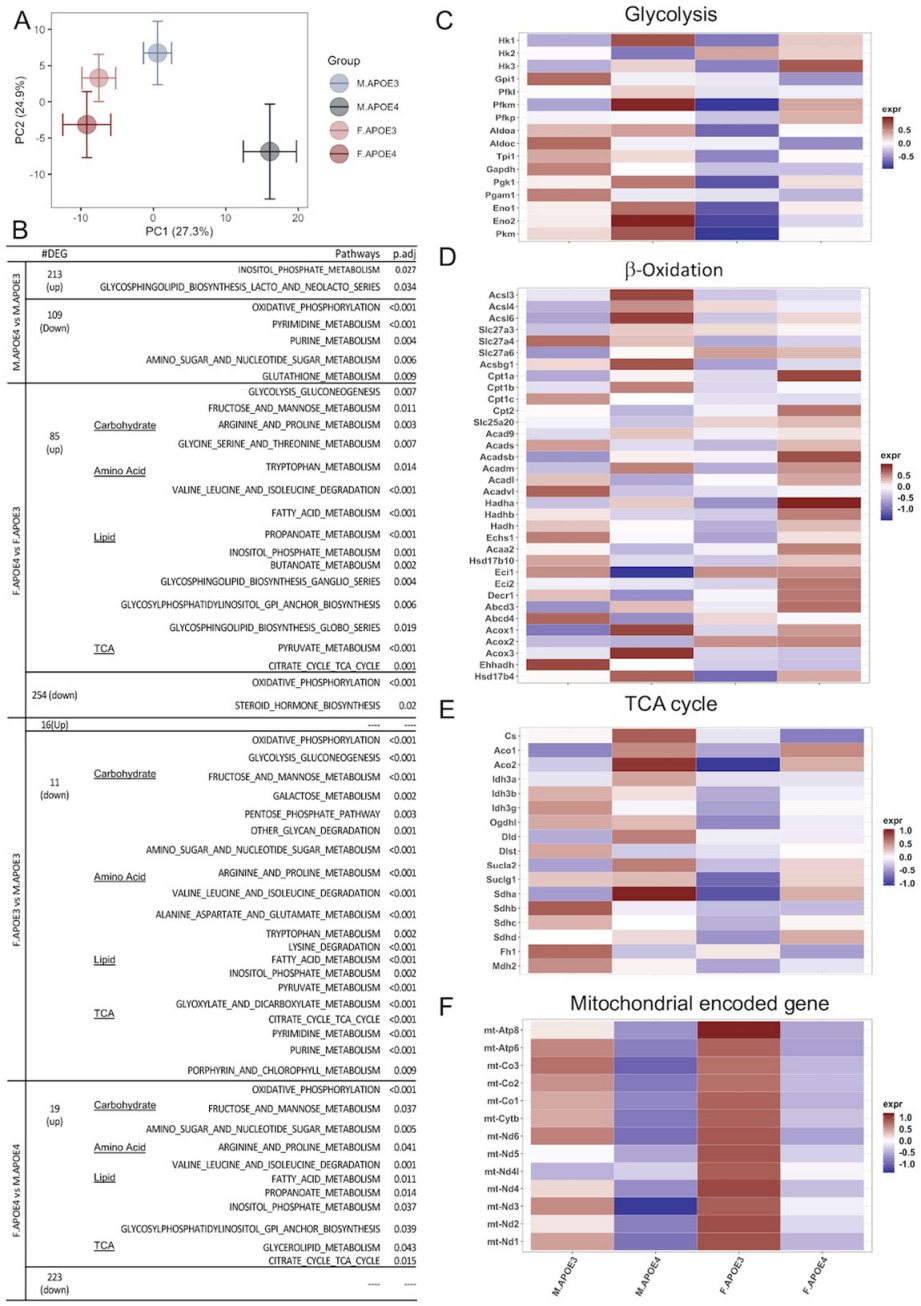
Pathway analysis of hippocampal transcriptome. (A) PCA analysis of RNA-Seq transcriptome on top 500 variance genes across all Groups. (B) Differentially Expressed Genes and GSEA enriched pathways between different sex and APOE genotypes. (C-E) Average heatmap of each sex and APOE genotype groups of genes directly involved in glycolysis, β-oxidation, and TCA cycle. (F) Average heatmap of mitochondrial encoded genes in each group showed lower mt-genes in APOE4 carriers comparing to their APOE3 compartments. Overall, males exhibited a transcriptome characterized by greater glycolytic and corresponding TCA transcriptome which was augmented in APOE4 males. In contrast, females exhibited a transcriptome characterized by expression of genes required for beta oxidation of lipids with a concomitant rise in subunits required for electron transport chain complex II.

To determine whether differences in metabolomic profile were paralleled by differences in gene expression, the transcriptome of the hippocampus of 16 months old mice were sequenced and analyzed. As with the metabolome in plasma and cortex, PCA indicated clustering by sex and APOE genotypes. PC1 clustered by sex whereas PC2 clustered by APOE genotype, suggesting that the transcriptome is also affected by sex and APOE genotypes. The asymmetric separation between sex and APOE further indicated an interaction between sex and APOE, with a greater difference between female and male in hAPOE4 carriers relative to hAPOE3 carriers (Fig 5A).

Consistent with PCA analysis, the total number of differentially expressed genes between male vs female hAPOE4 mice was greater compared to male vs female hAPOE3 mice (Fig 5B).

To pursue potential mechanisms underlying both sex and APOE genotype differences, transcriptomic pathway analyses were conducted. Gene Set Enrichment Analysis (GSEA) further highlighted differences in metabolic pathways by sex and APOE genotype. As shown in Fig 5B, lipid related pathways were significantly increased in hAPOE4 mice compared to hAPOE3 mice which was greatest in hAPOE4 females relative to hAPOE4 males. This is consistent with the metabolomic data, showing higher lipid levels in both hAPOE4 male and female brains, and high circulating acylcarnitine levels in hAPOE4 females (Figs 3 and 4).

If the brain is utilizing auxiliary fuels, such as lipids or amino acids, then the gene expression profile relevant to metabolism of each fuel could be a potential indicator of the energy source being utilized by brain. Transcriptomic profile in males indicated higher levels of amino acid metabolites (Fig 4B), glycolysis and TCA genes (Fig 5C and 5E) compared to hAPOE3 and hAPOE4 females. In contrast, female hAPOE4 carriers exhibited higher expression levels of acylcarnitine (Fig 4C) and β-oxidation related genes (Fig 5C), indicating a more lipid biology in female hAPOE4 carriers. Interestingly, female hAPOE3 carriers exhibited the lowest expression of all three metabolism pathways than either male hAPOE3 or female hAPOE4 carriers (Fig 5B–5E), which is consistent with the cortex metabolomic data, showing the lowest levels of amino acid, acylcarnitine, sphingomyelin and PC in female hAPOE3 carriers (Fig 4). Interestingly, in response to the metabolism pathway changes in metabolomics and transcriptomics, the hAPOE4 carriers exhibited systematically lower mitochondrial genome encoded genes than the hAPOE3 carriers in both males and females (Fig 5F).

### APOE and sex impacts antigen presentation and interferon response

Chromosomal sex and APOE genotype have distinct effects on lipid and amino acid metabolism and the corresponding bioenergetic profiles. Alterations in bioenergetic and metabolomic profiles in the brain can in turn impact neuroinflammation. Increased β-oxidation in hAPOE4 females and a concomitant increase in lysoPCs led us to hypothesize inflammatory pathways related to lipid metabolism would be affected.

**Fig 6:**
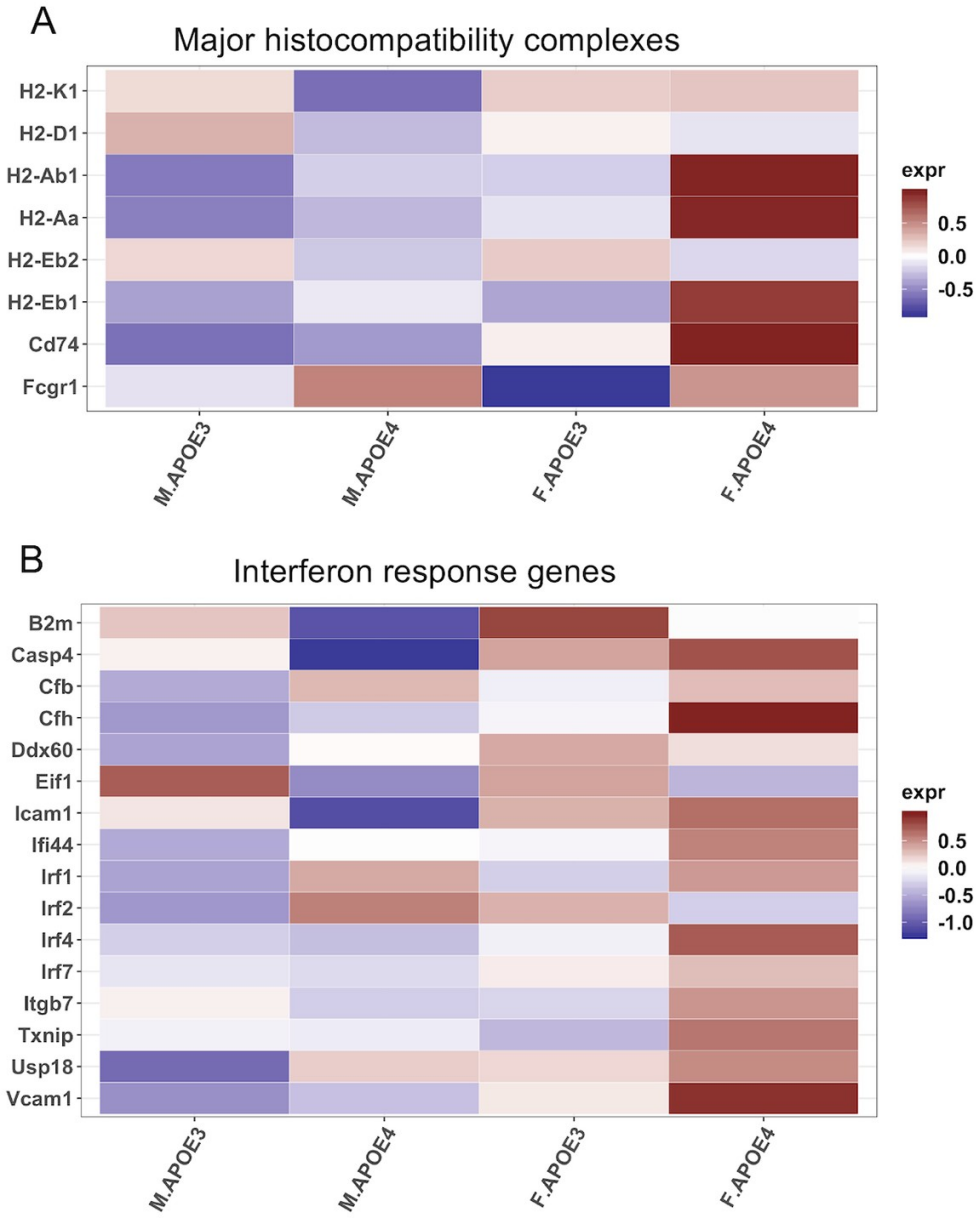
Transcriptomic analysis of major histocompatibility complex and interferon response in the hippocampus. (A) Major histocompatibility complexes I & II. (B) Interferon response genes.

To address this hypothesis, targeted differential gene expression analysis of inflammatory genes participating in myelin and lipid metabolism was conducted. Expression of transcripts for major histocompatibility complex and related genes (Fig 6A) and interferon response were increased in hAPOE4 females relative to hAPOE3 females and relative to both hAPOE3 and hAPOE4 males (Fig 6B). Increased major histocompatibility complex and interferon response gene expression is consistent with microglial reactivity and antigen presentation mediated by damage.

These data suggest that the combination of sex and APOE genotype affects metabolism, bioenergetics and neuroinflammation in tandem. Humanized APOE4 females present a phenotype that is robust in lipid metabolism and show a distinctively high expression of antigen presentation and interferon response genes, which may be contributing to microglial reactivity further exacerbating the at-risk aging phenotype.

### Impact of sex and APOE genotype regional brain volume and microstructural parameters

An increase in major histocompatibility complex and interferon response gene expression is consistent with microglial reactivity and antigen presentation mediated by damage associated molecular patterns. One potential contributor to activation of this network is debris generated by neuronal and or white matter degeneration. To address this hypothesis, T2-weighted and high-resolution diffusion-weighted imaging on perfused fixed skulls from hAPOE3 and hAPOE4 male and female mice at 16 months of age was conducted.

**Fig 7:**
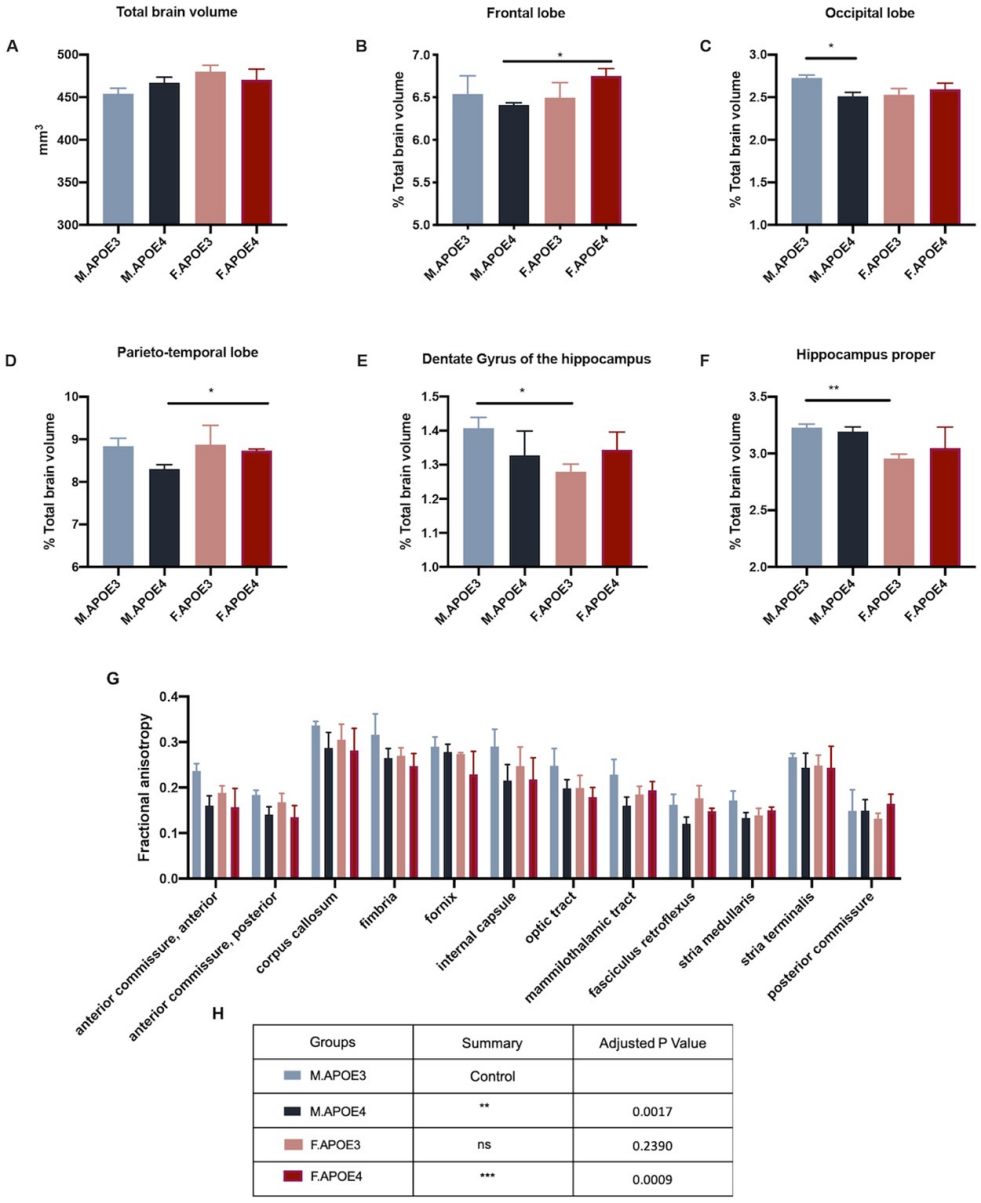
Impact of sex and APOE genotype on brain structural volume and diffusion metrics. Perfused fixed skulls of 16 months old APOE3 and APOE4 male and female mice underwent T2-weighted and diffusion-weighted imaging. T2-weighted imaging was used to conduct (A) Total brain volume measurements. (B-F) Parcellated brain regions normalized to total brain volume were plotted. (B) frontal lobe, (C) occipital lobe, (D) parieto-temporal lobe, (E) dentate gyrus of the hippocampus, (F) hippocampus proper. (G-H) Averaged value of the top-quartile of diffusion-metric fractional anisotropy for major white matter tracts(n = 2-3/group). (H) Friedman’s non-parametric rank test for fractional anisotropy of white matter tracts (G) in comparison to APOE3 males. Data presented indicate mean ± SEM, *p<0.05, **p<0.01.

While total brain volume was unaffected by sex and APOE genotype, as there were no significant differences observed (Fig 7A), regional brain volumes normalized to total brain volume were sensitive to sex and APOE genotype. Cortical regions were susceptible to sex differences in hAPOE4 mice as the frontal and parieto-temporal lobe were significantly smaller in hAPOE4 males than females (Fig 7B and 7D). The hippocampus was more susceptible to sex differences in hAPOE3 mice as the dentate gyrus of the hippocampus and hippocampus proper was smaller in female hAPOE3 mice (Fig 7E and 7F). Only the occipital lobe showed susceptibility to APOE genotype differences in males, with hAPOE4 animals being smaller (Fig 7C).

Diffusion-tensor metric fractional anisotropy (FA), an indicator of white matter integrity, was more susceptible APOE genotype difference. Humanized APOE3 males had significantly higher FA values than hAPOE4 males and females across different white matter tracts including internal capsule, fimbria, anterior commissure and corpus callosum (Fig 7G). Humanized APOE3 females also showed a similar trend.

Together, these data suggest that structural brain volume changes may be more susceptible to sex differences but microstructural changes in the white matter tracts are more sensitive to APOE genotype, particularly in hAPOE4 females, consistent with the cortical metabolomic profile.

### Cortical beta amyloid was detected in APOE4 mice

**Fig 8:**
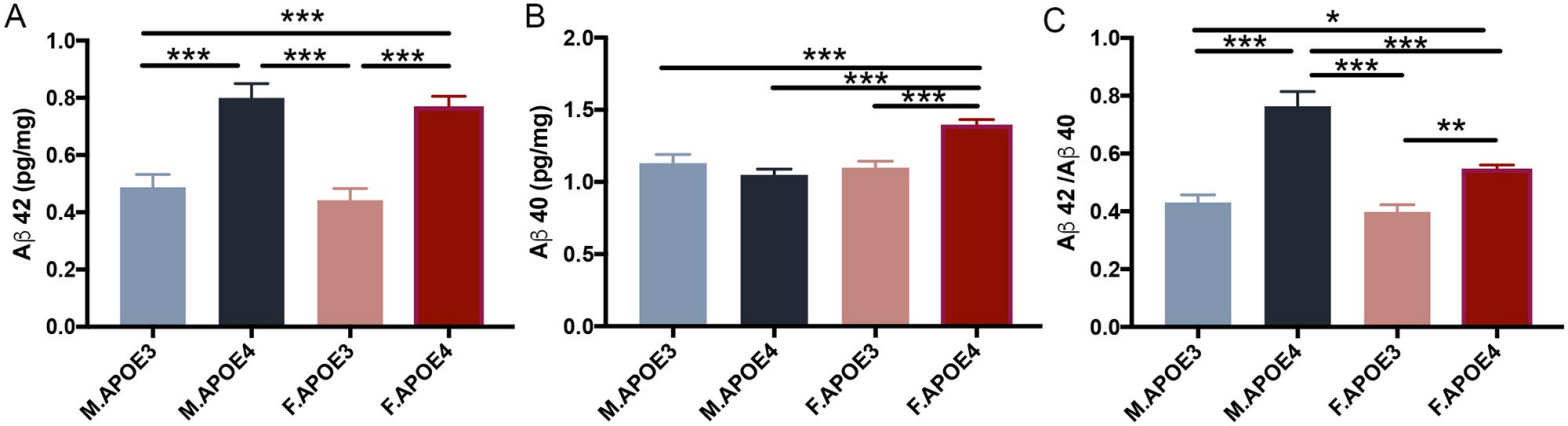
Cortical amyloid beta levels associate with APOE4 genotype in males and females. Cortical Aβ42 (A) level was significantly higher in APOE4 mice compared to APOE3 mice. APOE4 females also had significantly higher Aβ40 (B) level compared to the other 3 groups. The Aβ42/40 ratio (C) was significantly higher in APOE4 males compared to females, and the ratio in both sex of APOE4 mice were significantly higher than that in APOE3 mice. All data are presented as mean ± SEM, * P< 0.05, ** P< 0.01, *** P< 0.001.

Consistent with the APOE-associated metabolomic, transcriptomic and structural changes, amyloid beta 42 levels (Fig 8A, N = 5) were significantly higher in hAPOE4 mice compared to hAPOE3 mice. hAPOE4 females exhibited significantly higher Aβ 40 (Fig 8B, N = 5) levels compared to the other 3 groups. The Aβ 42/40 ratios (Fig 8C, N = 5) were significantly higher in hAPOE4 males compared to females which was driven by hAPOE4 male generation of Aβ 42 with no increase in Aβ 40 production whereas hAPOE4 females generated both Aβ 42 and 40. The Aβ 42/40 ratio in both male and female hAPOE4 mice were significantly higher than those in hAPOE3 mice. Together, these data indicated that the hAPOE4 isoform affected the beta amyloid pathogenesis by driving its generation in brain.

## Discussion

To investigate the systemic modulation of sex and APOE genotype, we performed a comprehensive analysis of both peripheral and brain metabolites, RNA transcripts in 16 months old hAPOE3 and hAPOE4 male and female mice. Results of these analyses indicate that lipid metabolism was upregulated in hAPOE4 mice, especially in females. Our results indicate that the impact of chromosomal sex is dominant in peripheral metabolism whereas the impact of APOE genotype is more apparent on brain metabolism. Generally, males are characterized by an amino acid metabolomic profile, while females are characterized by lipid metabolomic profile. The presence of hAPOE4 allele increased the sex-specific difference in peripheral metabolomic profile and resulted in significantly high levels of amino acids in hAPOE4 males and significantly high levels of acylcarnitine and sphingomyelins in hAPOE4 females when compared to the other three groups.

In addition, the transcriptomic profile in brain indicated that in hAPOE4 males, the glycolysis and TCA cycle pathways were upregulated compared to the other groups. Combined with the increased amino acids levels in both plasma and brain in hAPOE4 males, these results suggested a specific increase in oxidative metabolism in hAPOE4 males.

In contrast, in hAPOE4 females, the β-oxidation pathway is upregulated while the glycolysis and TCA cycle pathways are suppressed. Together with the fact that the plasma acylcarnitine levels are high but the brain plasma acylcarnitine levels are relatively low in these mice, suggest that mitochondrial oxidative phosphorylation is diminished in the hAPOE4 female brain which occurs in parallel to upregulation of lipid metabolism for use of lipids as an alternative fuel source. The brain transcriptomic profile of reduced expression of mitochondrial oxidative phosphorylation genes coupled with a rise in genes required for lipid metabolism may suggest an overall decline in brain bioenergetic capacity and a metabolic shift from glucose-derived fuels toward lipid catabolism and beta oxidation. This altered brain energy metabolism can be associated with the activation of neuroinflammation pathways especially involving antigen presentation and interferon response genes. These changes combined contribute to the development of APOE4-driven at-risk phenotype for AD in females.

Phosphatidylcholines (PCs), as the most abundant glycerophospholipids in brain, are key compositions of neural membrane and precursors for multiple lipid second messengers involved in intraneuronal signal transduction [80]. Alterations in PCs metabolism has been reported to correlate with and contribute to AD pathology [76]. Lower PC values [81] and increased lysoPC/PC ratio [82] have been reported in AD patients and lysoPCs is increasing recognized as a key mediator involved in oxidative stress and inflammatory responses [83] contributing to AD pathology. Our data demonstrated that relative to hAPOE3 mice, lysoPCs levels are higher in hAPOE4 mice, especially in the brain. Therefore, APOE4 associated alteration of PCs metabolism may contribute to APOE4-driven increase in AD pathology.

Altered bioenergetics and increased lipid metabolism as observed in hAPOE4 females is accompanied by the increased expression of interferon response genes and major histocompatibility complex genes possibly associated with the increased production of damage associated molecular patterns such as myelin debris [84]. Increased expression of major histocompatibility complexes has been associated with reactive microglial phenotype and with late stage disease progression of AD [85].

Given the system-wide changes affected by sex, APOE genotype and their interaction we anticipated myelin integrity and amyloid-β generation could also be affected. Consistent with clinical studies [86, 87], APOE4 females and males had lower fractional anisotropy across several white matter tracts indicating lower myelin integrity. Coupled with reduced myelin integrity, hAPOE4 animals showed an increased Aβ42 generation.

Findings of the contrast between the influence of chromosomal sex on peripheral metabolome vs APOE genotype effect on brain metabolome and transcriptome require further study and replication in other models and in human biological samples.

Collectively, these analyses indicate the broad impact of chromosomal sex and APOE genotype on metabolic profile, transcriptional networks in brain and on brain structure. From a translation prospective, the humanized APOE3 and APOE4 mouse model has consistencies with the human metabolome and in expression of hallmarks of Alzheimer’s pathology i.e. amyloid-β generation. Utilization of this model as a translationally valid therapeutic development is promising and requires further validation.

## Supporting information

S1 Table Concentrations of p180 metabolites in plasma. (XLSX)

S2 Table Concentrations of p180 metabolites in cortex. (XLSX)

## References

[1] Corder E, Saunders A, Strittmatter W, Schmechel D, Gaskell P, Small G, et al Gene dose of apolipoprotein E type 4 allele and the risk of Alzheimer’s disease in late onset families. Science. 1993;261(5123):921–3. 10.1126/science.8346443 8346443

[2] Poirier J, Bertrand P, Kogan S, Gauthier S, Davignon J, Bouthillier D. Apolipoprotein E polymorphism and Alzheimer’s disease. The Lancet. 1993;342(8873):697–9. 10.1016/0140-6736(93)91705-q 8103819

[3] Saunders AM, Strittmatter WJ, Schmechel D, George-Hyslop PS, Pericak-Vance MA, Joo S, et al Association of apolipoprotein E allele ϵ4 with late‐onset familial and sporadic Alzheimer’s disease. Neurology. 1993;43(8):1467-. 10.1212/wnl.43.8.1467 8350998

[4] Rebeck GW, Reiter JS, Strickland DK, Hyman BT. Apolipoprotein E in sporadic Alzheimer’s disease: allelic variation and receptor interactions. Neuron. 1993;11(4):575–80. 10.1016/0896-6273(93)90070-8 8398148

[5] Carrieri G, Bonafè M, De Luca M, Rose G, Varcasia O, Bruni A, et al Mitochondrial DNA haplogroups and APOE4 allele are non-independent variables in sporadic Alzheimer’s disease. Human genetics. 2001;108(3):194–8. 10.1007/s004390100463 11354629

[6] Maruszak A, Safranow K, Branicki W, Gaweda-Walerych K, Pospiech E, Gabryelewicz T, et al The impact of mitochondrial and nuclear DNA variants on late-onset Alzheimer’s disease risk. Journal of Alzheimer’s disease: JAD. 2011;27(1):197–210. Epub 2011/07/30. 10.3233/JAD-2011-110710 . 21799244

[7] Edland SD, Tobe VO, Rieder MJ, Bowen JD, McCormick W, Teri L, et al Mitochondrial genetic variants and Alzheimer disease: a case-control study of the T4336C and G5460A variants. Alzheimer disease and associated disorders. 2002;16(1):1–7. Epub 2002/03/08. 10.1097/00002093-200201000-00001 . 11882743

[8] Coto E, Gomez J, Alonso B, Corao AI, Diaz M, Menendez M, et al Late-onset Alzheimer’s disease is associated with mitochondrial DNA 7028C/haplogroup H and D310 poly-C tract heteroplasmy. Neurogenetics. 2011;12(4):345–6. Epub 2011/08/09. 10.1007/s10048-011-0295-4 . 21822896

[9] Brookmeyer R, Gray S, Kawas C. Projections of Alzheimer’s disease in the United States and the public health impact of delaying disease onset. American journal of public health. 1998;88(9):1337–42. Epub 1998/09/16. 10.2105/ajph.88.9.1337 . 9736873 PMC1509089

[10] Seshadri S, Beiser A, Kelly-Hayes M, Kase CS, Au R, Kannel WB, et al The lifetime risk of stroke: estimates from the Framingham Study. Stroke; a journal of cerebral circulation. 2006;37(2):345–50. Epub 2006/01/07. 10.1161/01.STR.0000199613.38911.b2 . 16397184

[11] Paganini-Hill A, Henderson VW. Estrogen deficiency and risk of Alzheimer’s disease in women. American journal of epidemiology. 1994;140(3):256–61. Epub 1994/08/01. 10.1093/oxfordjournals.aje.a117244 . 8030628

[12] Brinton RD. The healthy cell bias of estrogen action: mitochondrial bioenergetics and neurological implications. Trends in neurosciences. 2008;31(10):529–37. Epub 2008/09/09. 10.1016/j.tins.2008.07.003 . 18774188 PMC10124615

[13] Brookmeyer R, Evans DA, Hebert L, Langa KM, Heeringa SG, Plassman BL, et al National estimates of the prevalence of Alzheimer’s disease in the United States. Alzheimer’s & dementia: the journal of the Alzheimer’s Association. 2011;7(1):61–73. Epub 2011/01/25. 10.1016/j.jalz.2010.11.007 21255744 PMC3052294

[14] Riedel BC, Thompson PM, Brinton RD. Age, APOE and sex: Triad of risk of Alzheimer’s disease. The Journal of Steroid Biochemistry and Molecular Biology. 2016;160:134–47. 10.1016/j.jsbmb.2016.03.012 26969397 PMC4905558

[15] Blass JP. Brain metabolism and brain disease: is metabolic deficiency the proximate cause of Alzheimer dementia? Journal of neuroscience research. 2001;66(5):851–6. Epub 2001/12/18. 10.1002/jnr.10087 . 11746411

[16] Cunnane S, Nugent S, Roy M, Courchesne-Loyer A, Croteau E, Tremblay S, et al Brain fuel metabolism, aging, and Alzheimer’s disease. Nutrition (Burbank, Los Angeles County, Calif). 2011;27(1):3–20. Epub 2010/11/03. 10.1016/j.nut.2010.07.021 21035308 PMC3478067

[17] De Santi S, de Leon MJ, Rusinek H, Convit A, Tarshish CY, Roche A, et al Hippocampal formation glucose metabolism and volume losses in MCI and AD. Neurobiology of aging. 2001;22(4):529–39. Epub 2001/07/11. 10.1016/s0197-4580(01)00230-5 . 11445252

[18] Ishii K, Sasaki M, Kitagaki H, Yamaji S, Sakamoto S, Matsuda K, et al Reduction of cerebellar glucose metabolism in advanced Alzheimer’s disease. Journal of nuclear medicine: official publication, Society of Nuclear Medicine. 1997;38(6):925–8. Epub 1997/06/01. . 9189143

[19] Mosconi L, Berti V, Guyara-Quinn C, McHugh P, Petrongolo G, Osorio RS, et al Perimenopause and emergence of an Alzheimer’s bioenergetic phenotype in brain and periphery. PloS one. 2017;12(10):e0185926 10.1371/journal.pone.0185926 29016679 PMC5634623

[20] Mosconi L, Mistur R, Switalski R, Brys M, Glodzik L, Rich K, et al Declining brain glucose metabolism in normal individuals with a maternal history of Alzheimer disease. Neurology. 2009;72(6):513–20. Epub 2008/11/14. 10.1212/01.wnl.0000333247.51383.43 19005175 PMC2677512

[21] Mosconi L, De Santi S, Li J, Tsui WH, Li Y, Boppana M, et al Hippocampal hypometabolism predicts cognitive decline from normal aging. Neurobiology of aging. 2008;29(5):676–92. 10.1016/j.neurobiolaging.2006.12.008 17222480 PMC2430185

[22] Mosconi L. Glucose metabolism in normal aging and Alzheimer’s disease: Methodological and physiological considerations for PET studies. Clin Transl Imaging. 2013;1(4) 10.1007/s40336-013-0026-y . 24409422 PMC3881550

[23] Reiman EM, Caselli RJ, Chen K, Alexander GE, Bandy D, Frost J. Declining brain activity in cognitively normal apolipoprotein E epsilon 4 heterozygotes: A foundation for using positron emission tomography to efficiently test treatments to prevent Alzheimer’s disease. Proceedings of the National Academy of Sciences of the United States of America. 2001;98(6):3334–9. Epub 2001/03/15. 10.1073/pnas.061509598 11248079 PMC30654

[24] Reiman EM, Chen K, Alexander GE, Caselli RJ, Bandy D, Osborne D, et al Functional brain abnormalities in young adults at genetic risk for late-onset Alzheimer’s dementia. Proceedings of the National Academy of Sciences of the United States of America. 2004;101(1):284–9. Epub 2003/12/23. 10.1073/pnas.2635903100 14688411 PMC314177

[25] Reiman EM, Chen K, Alexander GE, Caselli RJ, Bandy D, Osborne D, et al Correlations between apolipoprotein E epsilon4 gene dose and brain-imaging measurements of regional hypometabolism. Proceedings of the National Academy of Sciences of the United States of America. 2005;102(23):8299–302. Epub 2005/06/04. 10.1073/pnas.0500579102 15932949 PMC1149416

[26] Mosconi L, Nacmias B, Sorbi S, De Cristofaro MT, Fayazz M, Tedde A, et al Brain metabolic decreases related to the dose of the ApoE e4 allele in Alzheimer’s disease. Journal of neurology, neurosurgery, and psychiatry. 2004;75(3):370–6. Epub 2004/02/18. 10.1136/jnnp.2003.014993 14966149 PMC1738980

[27] Mosconi L, Perani D, Sorbi S, Herholz K, Nacmias B, Holthoff V, et al MCI conversion to dementia and the APOE genotype: a prediction study with FDG-PET. Neurology. 2004;63(12):2332–40. Epub 2004/12/30. 10.1212/01.wnl.0000147469.18313.3b . 15623696

[28] Mosconi L, Sorbi S, Nacmias B, De Cristofaro MT, Fayyaz M, Bracco L, et al Age and ApoE genotype interaction in Alzheimer’s disease: an FDG-PET study. Psychiatry research. 2004;130(2):141–51. Epub 2004/03/23. 10.1016/j.pscychresns.2003.12.005 . 15033184

[29] Mosconi L, Herholz K, Prohovnik I, Nacmias B, De Cristofaro MT, Fayyaz M, et al Metabolic interaction between ApoE genotype and onset age in Alzheimer’s disease: implications for brain reserve. Journal of neurology, neurosurgery, and psychiatry. 2005;76(1):15–23. Epub 2004/12/21. 10.1136/jnnp.2003.030882 15607989 PMC1739315

[30] Mosconi L, De Santi S, Brys M, Tsui WH, Pirraglia E, Glodzik-Sobanska L, et al Hypometabolism and altered cerebrospinal fluid markers in normal apolipoprotein E E4 carriers with subjective memory complaints. Biological psychiatry. 2008;63(6):609–18. Epub 2007/08/28. 10.1016/j.biopsych.2007.05.030 17720148 PMC2386268

[31] Valla J, Yaari R, Wolf AB, Kusne Y, Beach TG, Roher AE, et al Reduced posterior cingulate mitochondrial activity in expired young adult carriers of the APOE epsilon4 allele, the major late-onset Alzheimer’s susceptibility gene. Journal of Alzheimer’s disease: JAD. 2010;22(1):307–13. Epub 2010/09/18. 10.3233/JAD-2010-100129 20847408 PMC3124564

[32] Wolf AB, Caselli RJ, Reiman EM, Valla J. APOE and neuroenergetics: an emerging paradigm in Alzheimer’s disease. Neurobiology of aging. 2013;34(4):1007–17. Epub 2012/11/20. 10.1016/j.neurobiolaging.2012.10.011 23159550 PMC3545040

[33] Mosconi L, Berti V, Quinn C, McHugh P, Petrongolo G, Varsavsky I, et al Sex differences in Alzheimer risk: Brain imaging of endocrine vs chronologic aging. Neurology. 2017;89(13):1382–90. Epub 2017/08/30. 10.1212/WNL.0000000000004425 . 28855400 PMC5652968

[34] Zhao L, Mao Z, Woody SK, Brinton RD. Sex differences in metabolic aging of the brain: insights into female susceptibility to Alzheimer’s disease. Neurobiology of aging. 2016;42:69–79. 10.1016/j.neurobiolaging.2016.02.011 27143423 PMC5644989

[35] Drzezga A, Riemenschneider M, Strassner B, Grimmer T, Peller M, Knoll A, et al Cerebral glucose metabolism in patients with AD and different APOE genotypes. Neurology. 2005;64(1):102–7. Epub 2005/01/12. 10.1212/01.WNL.0000148478.39691.D3 . 15642911

[36] Kish SJ, Mastrogiacomo F, Guttman M, Furukawa Y, Taanman JW, Dozic S, et al Decreased brain protein levels of cytochrome oxidase subunits in Alzheimer’s disease and in hereditary spinocerebellar ataxia disorders: a nonspecific change? Journal of neurochemistry. 1999;72(2):700–7. Epub 1999/02/04. 10.1046/j.1471-4159.1999.0720700.x . 9930743

[37] Chandrasekaran K, Giordano T, Brady DR, Stoll J, Martin LJ, Rapoport SI. Impairment in mitochondrial cytochrome oxidase gene expression in Alzheimer disease. Brain research Molecular brain research. 1994;24(1–4):336–40. Epub 1994/07/01. 10.1016/0169-328x(94)90147-3 . 7968373

[38] Aksenov MY, Tucker HM, Nair P, Aksenova MV, Butterfield DA, Estus S, et al The expression of several mitochondrial and nuclear genes encoding the subunits of electron transport chain enzyme complexes, cytochrome c oxidase, and NADH dehydrogenase, in different brain regions in Alzheimer’s disease. Neurochemical research. 1999;24(6):767–74. Epub 1999/08/14. 10.1023/a:1020783614031 . 10447460

[39] Maurer I, Zierz S, Moller HJ. A selective defect of cytochrome c oxidase is present in brain of Alzheimer disease patients. Neurobiology of aging. 2000;21(3):455–62. Epub 2000/06/20. 10.1016/s0197-4580(00)00112-3 . 10858595

[40] Parker WD, Filley CM, Parks JK. Cytochrome oxidase deficiency in Alzheimer’s disease. Neurology. 1990;40(8):1302 10.1212/wnl.40.8.1302 2166249

[41] Parker WD Jr., Parks J, Filley CM, Kleinschmidt-DeMasters BK. Electron transport chain defects in Alzheimer’s disease brain. Neurology. 1994;44(6):1090–6. Epub 1994/06/01. 10.1212/wnl.44.6.1090 . 8208407

[42] Yao J, Rettberg JR, Klosinski LP, Cadenas E, Brinton RD. Shift in brain metabolism in late onset Alzheimer’s disease: implications for biomarkers and therapeutic interventions. Molecular aspects of medicine. 2011;32(4–6):247–57. Epub 2011/10/26. 10.1016/j.mam.2011.10.005 22024249 PMC3658304

[43] Zhao L, Mao Z, Woody SK, Brinton RD. Sex differences in metabolic aging of the brain: insights into female susceptibility to Alzheimer’s disease. Neurobiology of aging. 2016;42:69–79. 10.1016/j.neurobiolaging.2016.02.011 . 27143423 PMC5644989

[44] Mosconi L. Perimenopause and emergence of an Alzheimer’s bioenergetic phenotype in brain and periphery. 2017;12(10). 10.1371/journal.pone.0185926 29016679 PMC5634623

[45] Yin F, Yao J, Sancheti H, Feng T, Melcangi RC, Morgan TE, et al The perimenopausal aging transition in the female rat brain: decline in bioenergetic systems and synaptic plasticity. Neurobiology of aging. 2015;36(7):2282–95. Epub 2015/04/30. 10.1016/j.neurobiolaging.2015.03.013 25921624 PMC4416218

[46] Gibson GE, Haroutunian V, Zhang H, Park LC, Shi Q, Lesser M, et al Mitochondrial damage in Alzheimer’s disease varies with apolipoprotein E genotype. Ann Neurol. 2000;48(3):297–303. Epub 2000/09/08. . 10976635

[47] Shi L, Du X, Zhou H, Tao C, Liu Y, Meng F, et al Cumulative effects of the ApoE genotype and gender on the synaptic proteome and oxidative stress in the mouse brain. The international journal of neuropsychopharmacology / official scientific journal of the Collegium Internationale Neuropsychopharmacologicum (CINP). 2014;17(11):1863–79. Epub 2014/05/09. 10.1017/s1461145714000601 . 24810422

[48] Xu PT, Li YJ, Qin XJ, Scherzer CR, Xu H, Schmechel DE, et al Differences in apolipoprotein E3/3 and E4/4 allele-specific gene expression in hippocampus in Alzheimer disease. Neurobiology of disease. 2006;21(2):256–75. Epub 2005/10/04. 10.1016/j.nbd.2005.07.004 . 16198584

[49] Xu PT, Li YJ, Qin XJ, Kroner C, Green-Odlum A, Xu H, et al A SAGE study of apolipoprotein E3/3, E3/4 and E4/4 allele-specific gene expression in hippocampus in Alzheimer disease. Molecular and cellular neurosciences. 2007;36(3):313–31. Epub 2007/09/08. 10.1016/j.mcn.2007.06.009 17822919 PMC3625967

[50] Chen HK, Ji ZS, Dodson SE, Miranda RD, Rosenblum CI, Reynolds IJ, et al Apolipoprotein E4 domain interaction mediates detrimental effects on mitochondria and is a potential therapeutic target for Alzheimer disease. The Journal of biological chemistry. 2011;286(7):5215–21. Epub 2010/12/02. 10.1074/jbc.M110.151084 21118811 PMC3037634

[51] Snowden SG, Ebshiana AA, Hye A, An Y, Pletnikova O, O’Brien R, et al Association between fatty acid metabolism in the brain and Alzheimer disease neuropathology and cognitive performance: A nontargeted metabolomic study. PLOS Medicine. 2017;14(3):e1002266 10.1371/journal.pmed.1002266 28323825 PMC5360226

[52] Montine TJ, Morrow JD. Fatty Acid Oxidation in the Pathogenesis of Alzheimer’s Disease. The American journal of pathology. 2005;166(5):1283–9 10.1016/S0002-9440(10)62347-4 15855630 PMC1606384

[53] Liu Q, Zhang J. Lipid metabolism in Alzheimer’s disease. Neuroscience bulletin. 2014;30(2):331–45. Epub 2014/04/15. 10.1007/s12264-013-1410-3 . 24733655 PMC5562656

[54] Klosinski LP, Yao J, Yin F, Fonteh AN, Harrington MG, Christensen TA, et al White Matter Lipids as a Ketogenic Fuel Supply in Aging Female Brain: Implications for Alzheimer’s Disease. EBioMedicine. 2015;2(12):1888–904. Epub 2016/02/05. 10.1016/j.ebiom.2015.11.002 26844268 PMC4703712

[55] Brinton RD. FUELING THE GLUCOSE-STARVED ALZHEIMER’S BRAIN: CATABOLISM OF WHITE MATTER IN THE BRAIN TO GENERATE KETONE BODIES. Alzheimer’s & Dementia: The Journal of the Alzheimer’s Association. 2017;13(7):P882–P3.

[56] Han X, D MH, McKeel DW Jr., Kelley J, Morris JC. Substantial sulfatide deficiency and ceramide elevation in very early Alzheimer’s disease: potential role in disease pathogenesis. Journal of neurochemistry. 2002;82(4):809–18. Epub 2002/10/03. 10.1046/j.1471-4159.2002.00997.x . 12358786

[57] Wood JA, Wood PL, Ryan R, Graff-Radford NR, Pilapil C, Robitaille Y, et al Cytokine indices in Alzheimer’s temporal cortex: no changes in mature IL-1 beta or IL-1RA but increases in the associated acute phase proteins IL-6, alpha 2-macroglobulin and C-reactive protein. Brain Res. 1993;629(2):245–52. Epub 1993/12/03. 10.1016/0006-8993(93)91327-o . 7509248

[58] Kang J, Rivest S. Lipid Metabolism and Neuroinflammation in Alzheimer’s Disease: A Role for Liver X Receptors. Endocrine Reviews. 2012;33(5):715–46. 10.1210/er.2011-1049 22766509

[59] Chong J, Yamamoto M, Xia J. MetaboAnalystR 2.0: From Raw Spectra to Biological Insights. Metabolites. 2019;9(3):57 10.3390/metabo9030057 30909447 PMC6468840

[60] Ritchie ME, Phipson B, Wu D, Hu Y, Law CW, Shi W, et al limma powers differential expression analyses for RNA-sequencing and microarray studies. Nucleic Acids Research. 2015;43(7):e47–e. 10.1093/nar/gkv007 25605792 PMC4402510

[61] Steadman PE, Ellegood J, Szulc KU, Turnbull DH, Joyner AL, Henkelman RM, et al Genetic effects on cerebellar structure across mouse models of autism using a magnetic resonance imaging atlas. Autism research: official journal of the International Society for Autism Research. 2014;7(1):124–37. Epub 2013/10/24. 10.1002/aur.1344 24151012 PMC4418792

[62] Jenkinson M, Beckmann CF, Behrens TE, Woolrich MW, Smith SM. FSL. NeuroImage. 2012;62(2):782–90. Epub 2011/10/08. 10.1016/j.neuroimage.2011.09.015 . 21979382

[63] Chen NK, Chang HC, Bilgin A, Bernstein A, Trouard TP. A diffusion-matched principal component analysis (DM-PCA) based two-channel denoising procedure for high-resolution diffusion-weighted MRI. PloS one. 2018;13(4):e0195952 Epub 2018/04/26. 10.1371/journal.pone.0195952 29694400 PMC5918820

[64] Smith DS, Li X, Arlinghaus LR, Yankeelov TE, Welch EB. DCEMRI.jl: a fast, validated, open source toolkit for dynamic contrast enhanced MRI analysis. PeerJ. 2015;3:e909 Epub 2015/04/30. 10.7717/peerj.909 25922795 PMC4411523

[65] Basser PJ, Mattiello J, LeBihan D. MR diffusion tensor spectroscopy and imaging. Biophysical journal. 1994;66(1):259–67. Epub 1994/01/01. 10.1016/S0006-3495(94)80775-1 8130344 PMC1275686

[66] Patro R, Duggal G, Love MI, Irizarry RA, Kingsford C. Salmon provides fast and bias-aware quantification of transcript expression. Nature Methods. 2017;14:417 https://www.nature.com/articles/nmeth.4197#supplementary-information. 28263959 10.1038/nmeth.4197 PMC5600148

[67] Soneson C, Love M, Robinson M. Differential analyses for RNA-seq: transcript-level estimates improve gene-level inferences [version 2; peer review: 2 approved]. F1000Research. 2016;4(1521). 10.12688/f1000research.7563.2 26925227 PMC4712774

[68] Love MI, Huber W, Anders S. Moderated estimation of fold change and dispersion for RNA-seq data with DESeq2. Genome biology. 2014;15(12):550 Epub 2014/12/18. 10.1186/s13059-014-0550-8 25516281 PMC4302049

[69] Subramanian A, Tamayo P, Mootha VK, Mukherjee S, Ebert BL, Gillette MA, et al Gene set enrichment analysis: A knowledge-based approach for interpreting genome-wide expression profiles. Proceedings of the National Academy of Sciences. 2005;102(43):15545–50. 10.1073/pnas.0506580102 16199517 PMC1239896

[70] Mootha VK, Lindgren CM, Eriksson K-F, Subramanian A, Sihag S, Lehar J, et al PGC-1α-responsive genes involved in oxidative phosphorylation are coordinately downregulated in human diabetes. Nature Genetics. 2003;34(3):267–73. 10.1038/ng1180 12808457

[71] Stephens M. False discovery rates: a new deal. Biostatistics. 2016;18(2):275–94. 10.1093/biostatistics/kxw041 27756721 PMC5379932

[72] Kanehisa M, Sato Y, Furumichi M, Morishima K, Tanabe M. New approach for understanding genome variations in KEGG. Nucleic Acids Res. 2019;47(D1):D590–d5. Epub 2018/10/16. 10.1093/nar/gky962 30321428 PMC6324070

[73] Kanehisa M, Goto S. KEGG: kyoto encyclopedia of genes and genomes. Nucleic Acids Res. 2000;28(1):27–30. Epub 1999/12/11. 10.1093/nar/28.1.27 10592173 PMC102409

[74] Mosconi L, Berti V, Swerdlow RH, Pupi A, Duara R, de Leon M. Maternal transmission of Alzheimer’s disease: Prodromal metabolic phenotype and the search for genes. Human Genomics. 2010;4(3):170 10.1186/1479-7364-4-3-170 20368139 PMC3033750

[75] Brinton RD, Yao J, Yin F, Mack WJ, Cadenas E. Perimenopause as a neurological transition state. Nat Rev Endocrinol. 2015;11(7):393–405. Epub 2015/05/27. 10.1038/nrendo.2015.82 . 26007613 PMC9934205

[76] Toledo JB, Arnold M, Kastenmuller G, Chang R, Baillie RA, Han X, et al Metabolic network failures in Alzheimer’s disease: A biochemical road map. Alzheimers Dement. 2017;13(9):965–84. Epub 2017/03/28. 10.1016/j.jalz.2017.01.020 28341160 PMC5866045

[77] St John-Williams L, Blach C, Toledo JB, Rotroff DM, Kim S, Klavins K, et al Targeted metabolomics and medication classification data from participants in the ADNI1 cohort. Sci Data. 2017;4:170140 Epub 2017/10/19. 10.1038/sdata.2017.140 29039849 PMC5644370

[78] Haughey NJ, Bandaru VV, Bae M, Mattson MP. Roles for dysfunctional sphingolipid metabolism in Alzheimer’s disease neuropathogenesis. Biochim Biophys Acta. 2010;1801(8):878–86. Epub 2010/05/11. 10.1016/j.bbalip.2010.05.003 20452460 PMC2907186

[79] Kosicek M, Hecimovic S. Phospholipids and Alzheimer’s disease: alterations, mechanisms and potential biomarkers. International journal of molecular sciences. 2013;14(1):1310–22. Epub 2013/01/12. 10.3390/ijms14011310 23306153 PMC3565322

[80] Farooqui AA, Horrocks LA, Farooqui T. Interactions between neural membrane glycerophospholipid and sphingolipid mediators: a recipe for neural cell survival or suicide. J Neurosci Res. 2007;85(9):1834–50. Epub 2007/03/30. 10.1002/jnr.21268 . 17393491

[81] Whiley L, Sen A, Heaton J, Proitsi P, Garcia-Gomez D, Leung R, et al Evidence of altered phosphatidylcholine metabolism in Alzheimer’s disease. Neurobiol Aging. 2014;35(2):271–8. Epub 2013/09/18. 10.1016/j.neurobiolaging.2013.08.001 24041970 PMC5866043

[82] Klavins K, Koal T, Dallmann G, Marksteiner J, Kemmler G, Humpel C. The ratio of phosphatidylcholines to lysophosphatidylcholines in plasma differentiates healthy controls from patients with Alzheimer’s disease and mild cognitive impairment. Alzheimers Dement (Amst). 2015;1(3):295–302. Epub 2016/01/09. 10.1016/j.dadm.2015.05.003 26744734 PMC4700585

[83] Law SH, Chan ML, Marathe GK, Parveen F, Chen CH, Ke LY. An Updated Review of Lysophosphatidylcholine Metabolism in Human Diseases. Int J Mol Sci. 2019;20(5). Epub 2019/03/09. 10.3390/ijms20051149 30845751 PMC6429061

[84] Mishra A, Brinton RD. Inflammation: Bridging Age, Menopause and APOEε4 Genotype to Alzheimer’s Disease. Frontiers in Aging Neuroscience. 2018;10(312). 10.3389/fnagi.2018.00312 30356809 PMC6189518

[85] Mathys H, Adaikkan C, Gao F, Young JZ, Manet E, Hemberg M, et al Temporal Tracking of Microglia Activation in Neurodegeneration at Single-Cell Resolution. Cell reports. 2017;21(2):366–80. Epub 2017/10/12. 10.1016/j.celrep.2017.09.039 29020624 PMC5642107

[86] Operto G, Cacciaglia R, Grau-Rivera O, Falcon C, Brugulat-Serrat A, Ródenas P, et al White matter microstructure is altered in cognitively normal middle-aged APOE-ε4 homozygotes. Alzheimer’s Research & Therapy. 2018;10(1):48 10.1186/s13195-018-0375-x 29793545 PMC5968505

[87] Honea RA, Vidoni E, Harsha A, Burns JM. Impact of APOE on the healthy aging brain: a voxel-based MRI and DTI study. Journal of Alzheimer’s disease: JAD. 2009;18(3):553–64. Epub 2009/07/09. 10.3233/JAD-2009-1163 19584447 PMC2892293

